# Identification of novel protein biomarkers for knee osteoarthritis by integrating human plasma proteome: evidence from Mendelian randomization and preliminary *in vitro* investigation

**DOI:** 10.3389/fmed.2026.1745114

**Published:** 2026-04-22

**Authors:** Wang Huang, WeiWei Hu, JiaYu Chen, Dan Yuan

**Affiliations:** 1 Qiannan Prefecture Traditional Chinese Medicine Hospital, Duyun, Guizhou, China; 2 Department of Orthopedics and Traumatology, Pingjiang County Hospital of Traditional Chinese Medicine, Yueyang, Hunan, China

**Keywords:** biomarkers, causal effect, knee osteoarthritis, Mendelian randomization, plasma protein

## Abstract

**Objective:**

This study sought to identify candidate plasma proteins with potential causal associations with knee osteoarthritis (KOA) through Mendelian randomization, and to provide preliminary biological evidence through *in vitro* experiments.

**Methods:**

Two-sample Mendelian randomization (MR) was conducted utilizing genetic instruments on 4,489 plasma proteins as exposures. Protein quantitative trait locus (pQTL) data were sourced from three large-scale studies. Genetic associations with KOA were obtained from a genome-wide association study (GWAS) of the European ancestry (accession ID: ebi-a-GCST007090; *n =* 403,124). The primary MR method was inverse variance weighting (IVW), supplemented by MR-Egger, weighted median, and weighted mode. Pleiotropy and reverse causality were assessed in sensitivity analyses, along with co-localization and protein–protein interaction (PPI) analyses. Preliminary *in vitro* exploration was conducted using macrophages (MØs) and synovial fibroblasts (SF) to assess biological plausibility.

**Results:**

Galectin-3 (encoded by LGALS3 [prot-b-6]) was identified as a biomarker of KOA (odds ratio [OR] = 1.07, 95% confidence interval [CI]:1.03–1.11, *p* = 0.00048), with moderate-to-strong co-localization support for a shared causal variant (posterior probability of H4 [PPH4] = 77.3%, lead single nucleotide polymorphism [SNP]: rs9323280). Exploratory *in vitro* experiments revealed that galectin-3 stimulation upregulated the expression of inflammatory cytokines (tumor necrosis factor alpha, interleukin-1β, and interleukin-18) and related inflammatory mediators (proteinase 3 [encoded by PRTN3] and myeloperoxidase [encoded by MPO]) in MØs, suggesting a potential pro-inflammatory association.

**Conclusion:**

This study identifies galectin-3 as a preferred plasma protein biomarker of KOA. The *in vitro* experiments provide preliminary biological plausibility for galectin-3-mediated inflammatory responses, suggesting potential involvement of myeloperoxidase and proteinase 3. These findings offer novel etiological insights and position galectin-3 as a therapeutic target, which warrants further exploration.

## 1 Introduction

Osteoarthritis (OA), a common degenerative joint disease, affects about 344 million individuals globally with soaring prevalence and disability rates, imposing a substantial global health burden (1). It predominantly targets weight-bearing joints. Consequently, knee osteoarthritis (KOA) becomes a major contributor to disability and a primary focus of research (2). The understanding of KOA has evolved from a mechanical “wear-and-tear” model to one centered on chronic low-grade inflammation, while synovitis is now known to drive the pathological changes of cartilage and bone (3). However, the transition to effective early intervention is hampered by the lack of specific molecular biomarkers (4), as clinical management often relies on late-stage joint replacement with variable outcomes (5).

Plasma proteins are promising biomarker candidates as they play a central role in biological processes and can indicate pathophysiological states (6). Enabled by high-throughput proteomics, genomic research has begun to map the genetic architecture of the plasma proteome (7). Observational studies have associated protein levels with the risk of OA (8, 9), yet no causality can be established for these associations. Therefore, a critical gap exists in the knowledge about the contribution of plasma proteins to the pathogenesis of KOA and the underlying regulatory pathways (10). It is essential to fill this gap and transform the findings into mechanistic insights and actionable targets (11).

Mendelian randomization (MR), a robust approach grounded in the principle of independent assortment, uses exposure-associated genetic variants as instrumental variables (IVs) for assessing exposure-outcome causality (12). Combined with proteomics, MR is a powerful tool for identifying novel biomarkers and drug targets in complex diseases (13). Two-sample MR further allows for the assessment of whether the causality is mediated by other factors (14). A key limitation is horizontal pleiotropy (genetic variants influencing outcomes via non-exposure pathways), which violates MR assumptions and requires further consideration (15). To clarify the causality, bidirectional MR (BMR) was used to explore reverse causality (whether outcomes drive exposures).

This study sought to identify specific plasma protein biomarkers causally linked to KOA utilizing MR, assess their biological plausibility through exploratory *in vitro* experiments, and investigate their potential associations with inflammation, thereby providing etiological insights for the diagnosis and treatment of KOA.

## 2 Methods

### 2.1 Data sources

Plasma protein data were derived from three independent studies: 3,622 proteins from an English population (6), 83 from a European population (16), and 1,124 from a German population (17), totaling 4,489 plasma proteins. KOA outcome data were obtained from the Integrative Epidemiology Unit Open GWAS project (IEU GWAS) database (ebi-a-GCST007090, *n =* 403,124) (18). All datasets were derived from European ancestry to minimize population stratification. All original studies obtained ethical approval and patient consent.

### 2.2 Selection of IVs

Single nucleotide polymorphisms (SNPs) linked to plasma proteins at genome-wide significance (*p* < 5 × 10^−8^) served as IVs. SNPs were clumped for linkage disequilibrium to ensure independence (LD: r^2^ < 0.001, kb = 10,000) (15). The selected IVs satisfied the three key MR assumptions: relevance (association with exposure), independence (no association with confounders), and exclusion restriction (influence on outcome only through the exposure) (19).

### 2.3 MR analyses

We performed bidirectional MR to evaluate the causal links between plasma proteins and KOA. The inverse variance weighting (IVW) method was adopted for the primary analysis (20). The MR-Egger, weighted median, and weighted mode methods were additionally employed to test the robustness of the results. To account for multiple testing across 4,489 proteins, a Bonferroni-corrected significance threshold of *p* < 1 × 10^−5^ was applied.

### 2.4 Consideration of statistical power

Although the large sample size provided sufficient statistical power, strict multiple testing corrections were still required to detect 4,489 proteins. To balance the stringency of correction with the exploratory goal of identifying potential biomarkers, a two-tiered significance threshold was applied. Proteins passing the Bonferroni-corrected threshold were deemed “significant,” while those with an IVW *p* < 0.01 were considered “critically significant” (21, 22) and retained for subsequent sensitivity and reverse MR analyses. This strategy enhanced the transparency of our analytical process by explicitly acknowledging and addressing the trade-off between false positives and statistical power in high-dimensional settings.

### 2.5 Sensitivity analysis

Comprehensive sensitivity analyses were conducted to address the potential bias and validate the robustness of MR results. For sensitivity analyses, the MR-Egger intercept test [*p* < 0.05 indicated significant pleiotropy (23)] and MR-PRESSO global test [*p* < 0.05 indicated overall pleiotropy; outlier SNPs were removed before re-analysis (24)] were performed to assess horizontal pleiotropy. Heterogeneity was evaluated utilizing Cochran’s Q statistic (*p* < 0.05 denoted significant heterogeneity, random-effects IVW was thus applied; otherwise, fixed-effects IVW was applied). Leave-one-out analysis (systematically excluding each SNP and repeating MR) was used to examine the influence of individual SNPs. To mitigate bias, population stratification was reduced by using only European-ancestry GWAS summary data (Table 1), with original studies applying standard genomic control for population structure. Inherent errors may be present in the measurement of plasma proteins and diagnosis of KOA. However, we collected data from well-established, high-quality consortia using standardized protocols, thereby minimizing these errors. A non-differential error would attenuate the causal estimates toward the null, leading to an underestimation of the true effect.

**Table 1:** Data sources of KOA and plasma proteome.

Variable	KOA	Plasma proteins		
Data sources	GWAS (ebi-a-GCST007090)	Sun et al. (6)	Folkersen et al. (16)	Suhre et al. (17)
Ethnicity	European	English	European	German
Year	2019	2018	2017	2017
*n*	Sample Size = 403,124	A total of 4,489 plasma proteins		

### 2.6 Reverse causality

The direction of the causal link between exposure and outcome can be ambiguous. Therefore, bidirectional MR (BMR) was used to evaluate both exposure-outcome and reverse relationships. Specifically, we swapped the exposure and outcome, treating KOA as the exposure and plasma proteins as the outcome, and repeated the two-sample MR using the same selection criteria for IVs as in the forward direction (25).

### 2.7 Co-localization analysis

Co-localization analysis was performed to assess whether the links between plasma proteins and KOA with genetic variants were due to shared causal genetic loci (26). Using the Bayesian R package “coloc,” the posterior probability of a shared causal variant (PPH4) was calculated. Evidence for co-localization was interpreted as follows: PPH4 ≥ 80% indicated strong evidence, 50% ≤ PPH4 < 80% indicated moderate evidence, and PPH4 < 50% indicated no evidence of co-localization (27, 28).

### 2.8 Protein–protein interaction (PPI) networks

The STRING database was leveraged to construct a PPI network to investigate the functional associations between proteins encoded by the identified candidate genes (29). Our study focused on interactions with a score of at least 0.4, which represented a moderate confidence network (30).

### 2.9 Replication cohort

To validate the robustness of the identification of galectin-3 (encoded by LGALS3), independent summary data from the FinnGen consortium (release 12) were utilized. Three outcomes were selected to cover different dimensions of the disease: (1) “Arthrosis_Knee” (broad definition, finngen_R12_M13_ARTHROSIS_KNEE); (2) “Primary_KOA” (strict definition, finngen_R12_PRIM_KNEEARTHROSIS); and (3) “Knee_Surgery” (severe outcome, finngen_R12_M13_ARTHROSIS_KNEE_PRIM_KNEESURG).

## 3 Experimental verification method

### 3.1 Cell culture and identification

#### 3.1.1 Cell culture

Rat peritoneal macrophages (MØs) (Cat. no. CP-R158) and rat synovial fibroblasts (SF) (Cat. no. CP-R329) were supplied by Procell Life Science & Technology Co., Ltd. (Wuhan, China). All cells were authenticated by the supplier and confirmed to be free of mycoplasma. They were placed in Dulbecco’s Modified Eagle Medium (DMEM), high glucose (Cat. no. PM150210, Procell, China) added with 10% fetal bovine serum (Cat. no. 164210, Procell, China) and 1% penicillin–streptomycin (Cat. no. PB180120, Procell, China) and incubated in a 5% CO2 incubator at 37 °C. Upon reaching 80–90% confluency, the cells were rinsed with PBS (Cat. no. G4202, Servicebio, China) and digested with 0.25% Trypsin–EDTA solution (Cat. no. G4001, Servicebio, China) for subculturing.

#### 3.1.2 Immunofluorescence identification of SFs

SFs (Cat. no. CP-R329) were seeded onto coverslips in 6-well plates. Upon reaching adequate density, the SFs were rinsed with PBS and fixed in 4% paraformaldehyde (Cat. no. G1101, Servicebio, China). Permeabilization was performed using 0.5% Triton X-100 (Cat. no. T8200, Solarbio, China). The cells were then added with a rabbit anti-vimentin antibody (1:500; Cat. no. ab92547, Abcam, UK) and incubated overnight at 4 °C. Subsequently, the cells were rinsed with PBS, added with a Cy3-conjugated goat anti-rabbit IgG secondary antibody (1:300; Cat. no. GB21303, Servicebio, China), and incubated for 1 h at room temperature in the dark. DAPI (Cat. no. G1012, Servicebio, China) was then used to counterstain the cellular nuclei. Images were captured with a fluorescence microscope.

#### 3.1.3 Flow cytometry identification of MØs

The purity of MØs (Cat. no. CP-R158) was verified by detecting CD68 expression using flow cytometry. The MØs were harvested and rinsed with PBS, then fixed and permeabilized using a Fixation/Permeabilization Kit (Cat. no. G1601, Servicebio, China). Subsequently, the cells were added with a rabbit anti-CD68 antibody (1:500; Cat. no. ab283654, Abcam, UK) and incubated for 30 min at 4 °C. Following this incubation, the cells were rinsed, added with an FITC-conjugated goat anti-rabbit IgG secondary antibody (1:200; Cat. no. GB22303, Servicebio, China), and incubated for 30 min in the dark. A flow cytometer (NovoCyte, ACEA Biosciences, China) was utilized for flow cytometry, and data were analyzed using NovoExpress software. The results showed that 91.9% of the cells were CD68-positive.

### 3.2 Grouping and intervention methods

MØs and SFs were divided into blank control groups (MØ1, SF1) and Galectin-3 stimulation groups (MØ2, SF2). The control groups were cultured in normal media for 24 h. The stimulation groups were treated with recombinant galectin-3 protein (Cat. no. HY-P70309, MedChemExpress, USA) for 24 h. To exclude potential interference from endotoxin contamination, a high-purity protein certified to have an endotoxin level of less than 1.0 EU/μg, as determined by the limulus amebocyte lysate (LAL) assay, was used. Cell pellets and supernatants were collected.

### 3.3 Enzyme-linked immunosorbent assay (ELISA) detection of TNF-*α*, IL-1β, and IL-18

ELISA kits supplied by Solarbio Science & Technology Co., Ltd. (Beijing, China), with catalog numbers SEKR-0009 (for rat TNF-α), SEKR-0002 (for rat IL-1β), and SEKR-0054 (for rat IL-18), were utilized to determine tumor necrosis factor alpha (TNF-α), interleukin-1β (IL-1β), and interleukin-18 (IL-18) levels in the cell culture supernatants. All steps were carried out per the manufacturer’s instructions. A microplate reader was utilized for measuring absorbance.

### 3.4 Western blot (WB) analysis

According to the grouping described in section 3.2, after collecting the cells, total protein was extracted utilizing RIPA lysis buffer (Cat. no. G2002, Servicebio, Wuhan, China) added with proteinase and phosphatase inhibitors. TA BCA protein assay kit (Cat. no. G2026, Servicebio, China) was used to measure protein concentration in the supernatant. The protein solution was mixed with a 5 × sodium dodecyl-sulfate polyacrylamide gel electrophoresis (SDS-PAGE) protein loading buffer (Cat. no. G2013, Servicebio, China) at a ratio of 4:1 and then heated in a 95 °C water bath for 10 min to denature the proteins, which were then separated by SDS-PAGE using a fast gel preparation kit (Cat. no. G2003, Servicebio, China) and transferred onto polyvinylidene fluoride membranes. The membranes were blocked with 5% non-fat milk for 1 h and incubated overnight at 4 °C with primary antibodies: rabbit anti-NF-κB p65 (phospho S536) (1:2,000; Cat. no. ab239882, Abcam, UK), mouse anti-PRTN3 (1:1,000; Cat. no. sc-74534, Santa Cruz Biotechnology, USA), mouse anti-MPO (1:1,000; Cat. no. sc-390109, Santa Cruz Biotechnology, USA), and rabbit anti-GAPDH (1:2,000; Cat. no. GB11002, Servicebio, China). Subsequently, the membranes were rinsed and then incubated with HRP-conjugated goat anti-rabbit or goat anti-mouse IgG secondary antibodies (1:5,000; Servicebio, China), depending on the host species of the primary antibodies, for 1 h at room temperature. An ECL chemiluminescence kit (Cat. no. G2014, Servicebio, China) was utilized to visualize the protein bands, whose grayscale values were generated in the ImageJ software (NIH, USA).

## 4 Statistical analysis and ethics

The “TwoSampleMR” and “MR-PRESSO” packages in R v4.4.2 were employed to perform all two-sample MR analyses (31, 32). Effect estimates were described as odds ratio (OR) with 95% confidence interval (CI). The strength of genetic instruments and potential weak instrument bias was evaluated by calculating the F-statistic for each SNP with the formula F = *β*^2^/SE^2^, where β denotes the effect size of the SNP on protein levels, and SE denotes the standard error. An F-statistic greater than 10 is generally considered to indicate sufficient instrument strength. Additionally, the proportion of variance explained by each instrument (R^2^) was estimated with the formula R^2^ = 2 × EAF × (1 − EAF) × β^2^, where EAF denotes the effect allele frequency. All *in vitro* experiments were independently repeated in at least three biological replicates (*n =* 3). Data were reported as mean ± standard deviation (SD). Comparisons between control and galectin-3 groups were performed using the two-tailed, unpaired Student’s *t*-test. GraphPad Prism v8.0 served as the statistical analysis tool. No additional ethical approval was necessary for this study, since it exclusively utilized open-access GWAS summary data. The original GWAS studies had already obtained appropriate ethical approvals.

## 5 Results

### 5.1 Identification of plasma proteins associated with KOA

The analysis was conducted using 4,489 plasma proteins as exposures. These proteins were derived from three studies: Benjamin et al.’s study, Folkersen et al.’s study, and Suhre et al.’s study. Outcome data were acquired from the IEU GWAS database for KOA (ebi-a-GCST007090, *n =* 403,124). These proteins were screened based on the following criteria: (1) SNPs that were significant in GWAS (*p* < 5 × 10^−8^), and (2) independent IVs with LD (r^2^ < 0.001, kb = 10,000) to ensure that the chosen genetic variations were independent (Supplementary Table S1). After applying Bonferroni correction (IVW’s *p* < 1 × 10^−5^), a significant association was found for 1 protein (Supplementary Table S2). Due to the limited number of positive findings after correction, proteins with an IVW *p* < 0.01 were selected to avoid loss of potentially significant results, yielding 60 critically significant proteins (Supplementary Table S3). After excluding those with inconsistent trends in the four MR models (inverse variance weighted, MR Egger, weighted median, and weighted mode), 13 critically significant proteins were retained (Supplementary Table S4).

**Figure 1:**
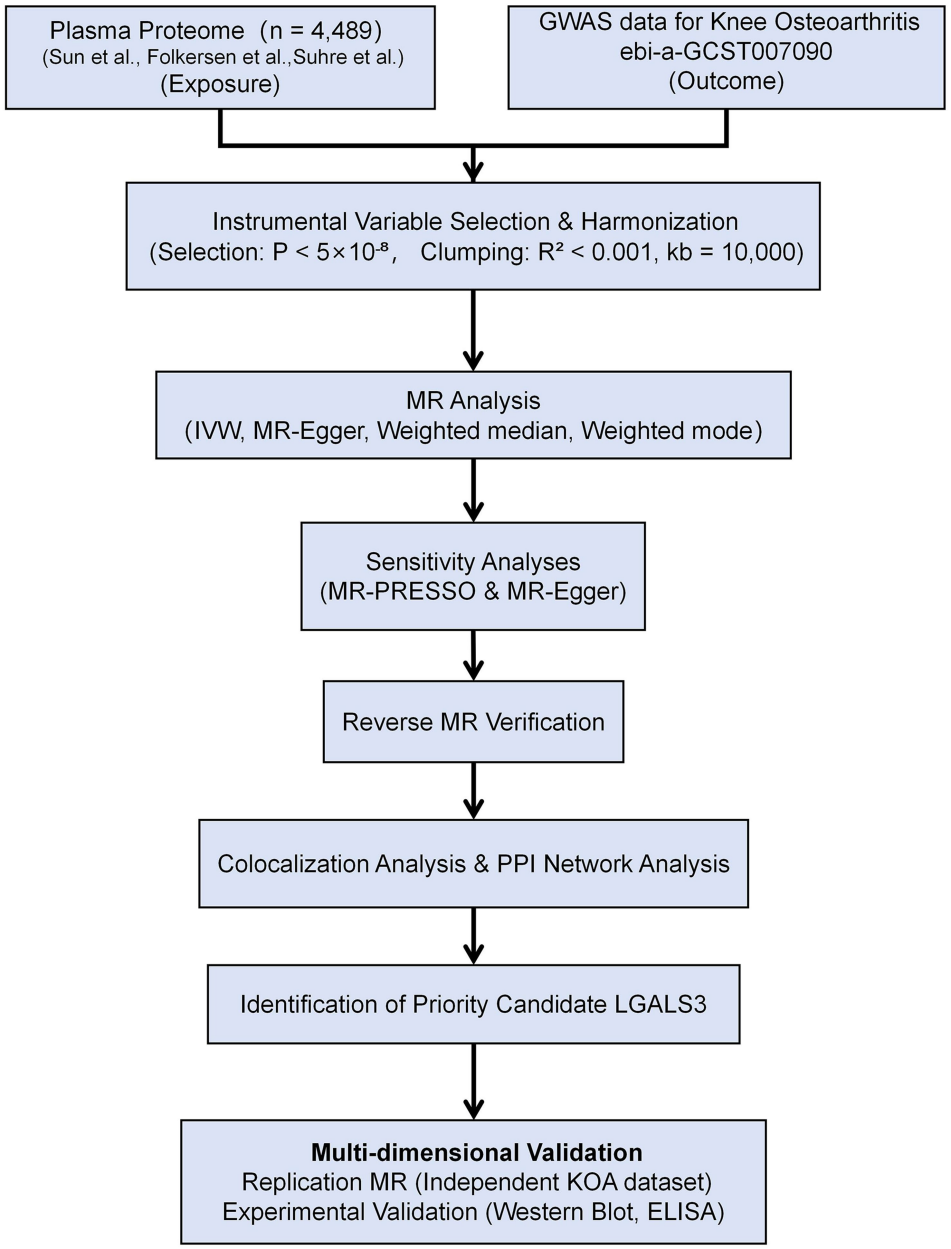
Flowchart.

To ensure adequate statistical power of these genetic instruments and mitigate potential weak instrument bias, we rigorously assessed the strength of each instrument with standard formulas, applying a stringent inclusion criterion for strong genetic instruments (*p* < 5 × 10^−8^). The F-statistic was calculated as F = β^2^/SE^2^, and R^2^ was calculated as R^2^ = 2 × EAF × (1 − EAF) × β^2^. The resulting F-statistics for the valid instruments ranged from 34.945 to 775.774, well above the conventional threshold of 10, which confirmed that our analysis was robust against weak instrument bias. Additionally, the variance explained R^2^ values ranged from 0.009 to 0.432, further supporting their predictive strength (Supplementary Table S5). Detailed screening and exclusion criteria are presented in Figure 1.

### 5.2 Sensitivity analyses

To ensure reliability of our primary findings, comprehensive sensitivity analyses were conducted for all candidate proteins. The results demonstrated robustness of the analysis results for the key candidate protein, galectin-3, with no evidence of marked bias. The MR-Egger intercept test showed no detectable horizontal pleiotropy (*p* = 0.395), and Cochran’s Q test revealed no marked heterogeneity (*p* = 0.227). The Steiger directionality test confirmed the correct direction of causality (*p* < 0.001), and leave-one-out analysis indicated that no single SNP dominated the association. Visualization by scatter plots, funnel plots, and leave-one-out plots (Supplementary Figure S1) further validated the stability of the results. Sensitivity analyses for the remaining candidate proteins also supported the robustness of their respective associations. Detailed results are provided in Supplementary Table S6.

### 5.3 Reverse causality verification

**Figure 2:**
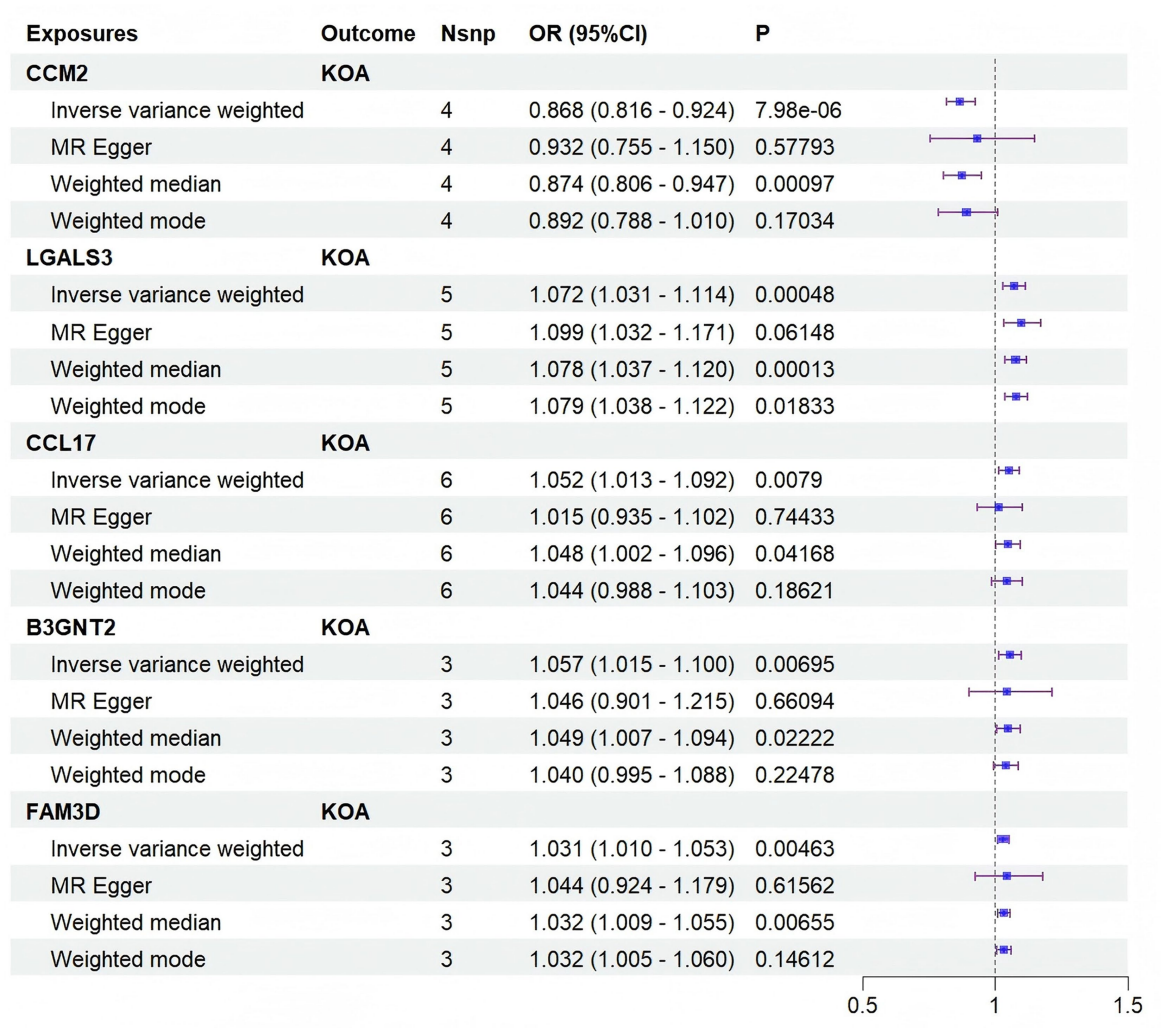
Forest plot of 5 plasma proteins with significant causal associations with KOA phenotypes.

Based on the above results, reverse verification was conducted on 14 proteins, including 1 significant protein and 13 critically significant proteins. Proteins that showed reverse causal effects, as indicated by an IVW *p*-value < 0.05 (see Supplementary Table S7), were excluded. As a result, 1 significant protein and 4 critically significant proteins were retained (Supplementary Table S8). CCM2 (prot-a-412) was identified as a protective factor for KOA (OR = 0.87, 95% CI: 0.82–0.92, *p* = 7.98 × 10^−6^), whereas LGALS3 (prot-b-6), CCL17 (prot-a-394), B3GNT2 (prot-a-213), and FAM3D (prot-a-1051) were identified as risk factors for KOA (LGALS3 [OR = 1.07, 95% CI: 1.03–1.11, *p* = 0.00048], CCL17 [OR = 1.05, 95% CI: 1.01–1.10, *p* = 0.00790], B3GNT2 [OR = 1.06, 95% CI: 1.02–1.10, *p* = 0.00695], and FAM3D [OR = 1.03, 95% CI: 1.01–1.05, *p* = 0.00463]). The causal estimates for proteins encoded by these 5 genes are shown in Figure 2.

### 5.4 Candidate prioritization

To address the issue of “threshold drift” and false-positive results, all tested proteins were corrected for false discovery rate (FDR). A complete set of q-values are reported in Supplementary Table S1. Among the five identified proteins, only galectin-3 (encoded by LGALS3, prot-b-6) was identified as the candidate protein with suggestive significance (q = 0.06631 < 0.1). Importantly, Sun et al. (6) confirmed that LGALS3 (prot-b-6) exhibits stable cis-genetic regulation. To further validate this point, genomic data from Open GWAS^1^ and the National Center for Biotechnology Information (NCBI)^2^ were used to map genomic coordinates. The lead SNP for LGALS3, rs9323280 (chr14: 55,334,969), is located approximately 190 kb from the LGALS3 locus (chr14: 55,129,252–55,145,430), confirming its status as a strong cis-pQTL (within the ±500 kb window), as detailed in Supplementary Figure S2.

Meanwhile, the NHGRI-EBI GWAS Catalog was used to detect potential associations between our lead instrumental variable rs9323280 and major potential confounders, with results detailed in Supplementary Table S11. Our analysis confirmed a significant association of rs9323280 with galectin-3 level (*p* = 7 × 10^−94^) (16), which verified the strength of the genetic instruments. Moreover, no genome-wide significant associations were observed between rs9323280 and risk factors for KOA (bone mineral density, obesity, blood lipid, and inflammatory markers). Furthermore, the only other trait found to be significantly associated with KOA was reticulocyte count (*p* = 2 × 10^−12^) (33), which is usually not considered a risk factor for KOA.

Given both the statistical differences and biological significance, galectin-3 was prioritized as the primary target for subsequent functional validation.

### 5.5 Co-localization analysis for identifying proteins associated with KOA

The coloc package in R was utilized to perform Bayesian co-localization analysis. Five hypotheses (H0–H4) were tested to reckon the probability of shared causal variants between plasma protein levels and KOA (26).

**Figure 3:**
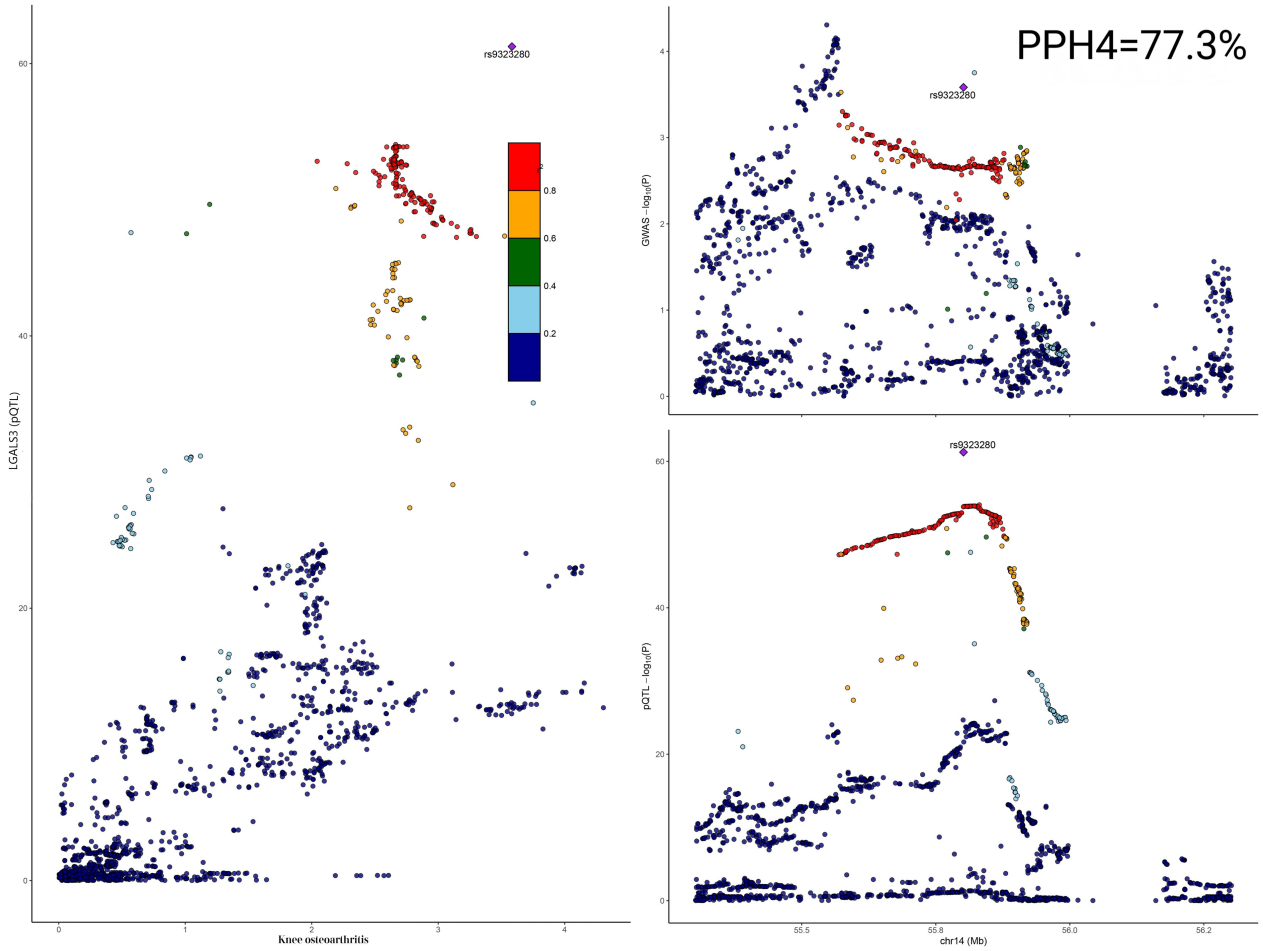
Co-localization analysis of galectin-3 protein and KOA.

Among the prioritized candidate genes, the association of galectin-3 with KOA appeared to be driven by an SNP (rs9323280), with a co-localization posterior probability of H4 (PPH4) of 77.3% (Figure 3; Supplementary Table S9). Although this value was slightly below our predefined threshold for strong evidence (PPH4 > 80%) (26), it exceeded the threshold commonly adopted in high-impact genomic studies recently published in journals such as Nature Communications, where PPH4 > 70% was considered evidence of co-localization (34, 35).

Comprehensive sensitivity analyses were performed to ensure this finding is robust. First, the genomic window size around the lead SNP was changed (from ±100 kb to ±500 kb), which yielded consistent PPH4 estimates and ruled out the influence of boundary deviation (Supplementary Table S10). Second, the sensitivity plot for prior probabilities demonstrated that the co-localization hypothesis (H4) was still supported across a range of plausible priors near the default setting (p12 = 10^−5^) (Supplementary Figure S3). Furthermore, regional visualization confirmed perfect alignment between the pQTL and GWAS signals at the LGALS3 locus, with consistent LD structures (Figure 3).

Collectively, these results provide moderate-to-strong evidence for a shared genetic basis underlying plasma galectin-3 levels and susceptibility to KOA, warranting further functional investigation.

### 5.6 Independent validation in FinnGen

The causal effect of galectin-3 on KOA was validated in an independent FinnGen dataset using different case definitions. Genetically predicted high plasma galectin-3 levels were consistently linked to heightened risks of KOA under a broad definition (Arthrosis_Knee: IVW OR = 1.06, 95% CI: 1.02–1.11, *p* = 0.00501). This association remained significant when a strict definition (Primary_KOA: IVW OR = 1.06, 95% CI: 1.01–1.12, *p* = 0.02900) or a severity-based definition (Knee_Surgery: IVW OR = 1.07, 95% CI: 1.02–1.12, *p* = 0.00869) was applied. The consistency of these results across alternative definitions strengthened the external validity of our findings. Detailed validation results are presented in Table 2.

**Table 2:** FinnGen_replication_results.

Exposure	Outcome	Method	Nsnp	b	SE	*p*-value	OR	OR_LCI	OR_UCI	
galectin 3 || id:prot-b-6	Arthrosis_Knee || finngen_R12_M13_ARTHROSIS_KNEE	Inverse variance weighted	5	0.0627436563572327	0.0223554404010189	0.00500612710716099	1.06475386136126	1.01910724411336	1.11244502659781	
galectin 3 || id:prot-b-6	Arthrosis_Knee || finngen_R12_M13_ARTHROSIS_KNEE	Weighted median	5	0.0517169057668378	0.0151635859034251	0.000648204278575807	1.05307758012132	1.02223997906885	1.08484544966079	
galectin 3 || id:prot-b-6	Arthrosis_Knee || finngen_R12_M13_ARTHROSIS_KNEE	MR Egger	5	0.0333293823262455	0.0377832107258538	0.442668397265282	1.03389102859472	0.960092436920893	1.11336223253337	
galectin 3 || id:prot-b-6	Primary_KOA || finngen_R12_PRIM_KNEEARTHROSIS	Inverse variance weighted	5	0.0581007950804165	0.0266099123219211	0.0290043958628205	1.05982181515185	1.00596307570107	1.11656412347835	
galectin 3 || id:prot-b-6	Primary_KOA || finngen_R12_PRIM_KNEEARTHROSIS	Weighted median	5	0.0447558921778672	0.0219193228104581	0.041166761805694	1.04577254748909	1.00179562968507	1.09167996812446	
galectin 3 || id:prot-b-6	Primary_KOA || finngen_R12_PRIM_KNEEARTHROSIS	MR Egger	5	0.0093780317829822	0.0378589780662291	0.820345499206096	1.00942214330831	0.93723093478496	1.08717395636853	
galectin 3 || id:prot-b-6	Knee_Surgery || finngen_R12_M13_ARTHROSIS_KNEE_PRIM_KNEESURG	Inverse variance weighted	5	0.0659290509255301	0.0251250455715766	0.00868943275157886	1.06815093015763	1.01682391614136	1.12206881789943	
galectin 3 || id:prot-b-6	Knee_Surgery || finngen_R12_M13_ARTHROSIS_KNEE_PRIM_KNEESURG	Weighted median	5	0.050068732965702	0.0205937731746429	0.0150465823751625	1.05134335583951	1.00975218316468	1.0946476475086	
galectin 3 || id:prot-b-6	Knee_Surgery || finngen_R12_M13_ARTHROSIS_KNEE_PRIM_KNEESURG	MR Egger	5	0.00861635952946595	0.0288191364109522	0.784469857427548	1.00865358720075	0.953258514856397	1.06726773809748	

### 5.7 Bioinformatic exploration of functional links among candidate proteins

**Figure 4:**
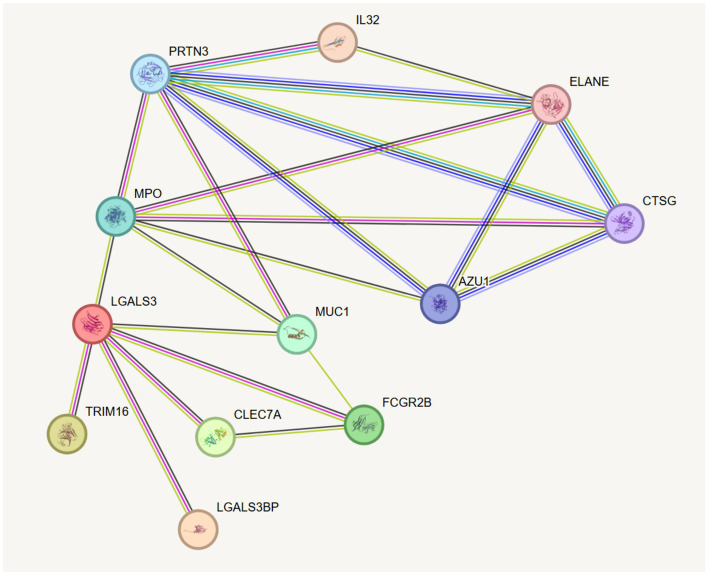
Exploratory protein–protein interaction (PPI) network visualization suggesting a potential functional link between galectin-3 and inflammatory mediators.

To elucidate the functional relationships among candidate proteins, the STRING^3^ database was leveraged to construct a PPI network (30). This network revealed a highly interconnected module centered on galectin-3 (Figure 4; Supplementary Table S12). KEGG pathway analysis further implicated these proteins in the key immunoregulatory processes.

Galectin-3 (encoded by LGALS3), a chimeric galectin expressed in immune cells, is a known mediator of inflammation and a marker for chondrocyte and osteoblast differentiation (36). Previous studies have indicated that it may facilitate the phosphoinositide 3-kinase (PI3K)/Akt signaling pathway, stimulating the synthesis of inflammatory mediators like IL-6 and nitric oxide in the synovium, potentially promoting osteoclast differentiation, chondrocyte apoptosis, and cartilage breakdown (37, 38).

Proteinase 3 (encoded by PRTN3) and myeloperoxidase (encoded by MPO) are neutrophil-derived proteins and autoantigens involved in vasculitis (39). Proteinase 3 is reported to amplify inflammation by cleaving and activating potent cytokines, including TNF-*α*, IL-1β, and IL-18, which exacerbate tissue damage (40).

Collectively, our integrated co-localization and PPI analyses uncovered a potential LGALS3-MPO-PRTN3 inflammatory module, providing a bioinformatic basis for further investigation into its role in the pathogenesis of KOA.

### 5.8 Exploratory *in vitro* investigation of galectin-3-associated inflammatory responses

#### 5.8.1 Phenotypic identification of MØs and SFs

Increasing evidence suggests that MØs produce various inflammatory cytokines (e.g., TNF-α and IL-1β), contributing to the pathogenesis of KOA. Moreover, these cytokines also further activate MØs, creating a proinflammatory cycle.

**Figure 5:**
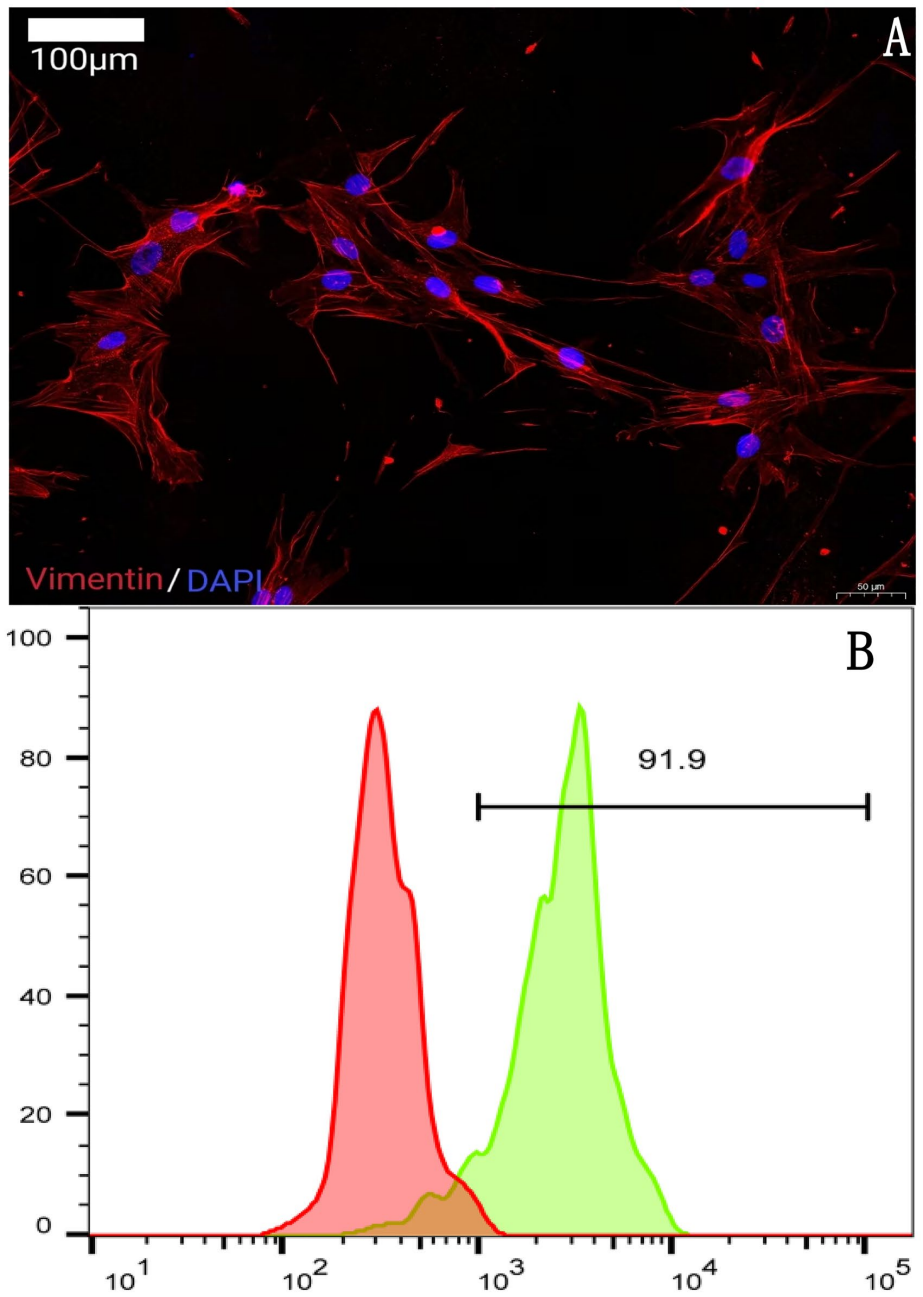
**(A)** Synovial fibroblast vimentin immunofluorescence detection results; **(B)** macrophage CD68 flow cytometry detection results.

SFs, as the primary cellular component of the synovium, play a crucial role in synovial inflammation, bone destruction, and pain associated with KOA. Therefore, these two cell types were selected to investigate the potential involvement of galectin-3-related signaling. Immunofluorescence staining of vimentin was performed to identify SFs (Figure 5A), and flow cytometry for CD68 was conducted to identify MØs (Figure 5B).

#### 5.8.2 Effect of galectin-3 on pro-inflammatory cytokine levels in cell supernatants

**Figure 6:**
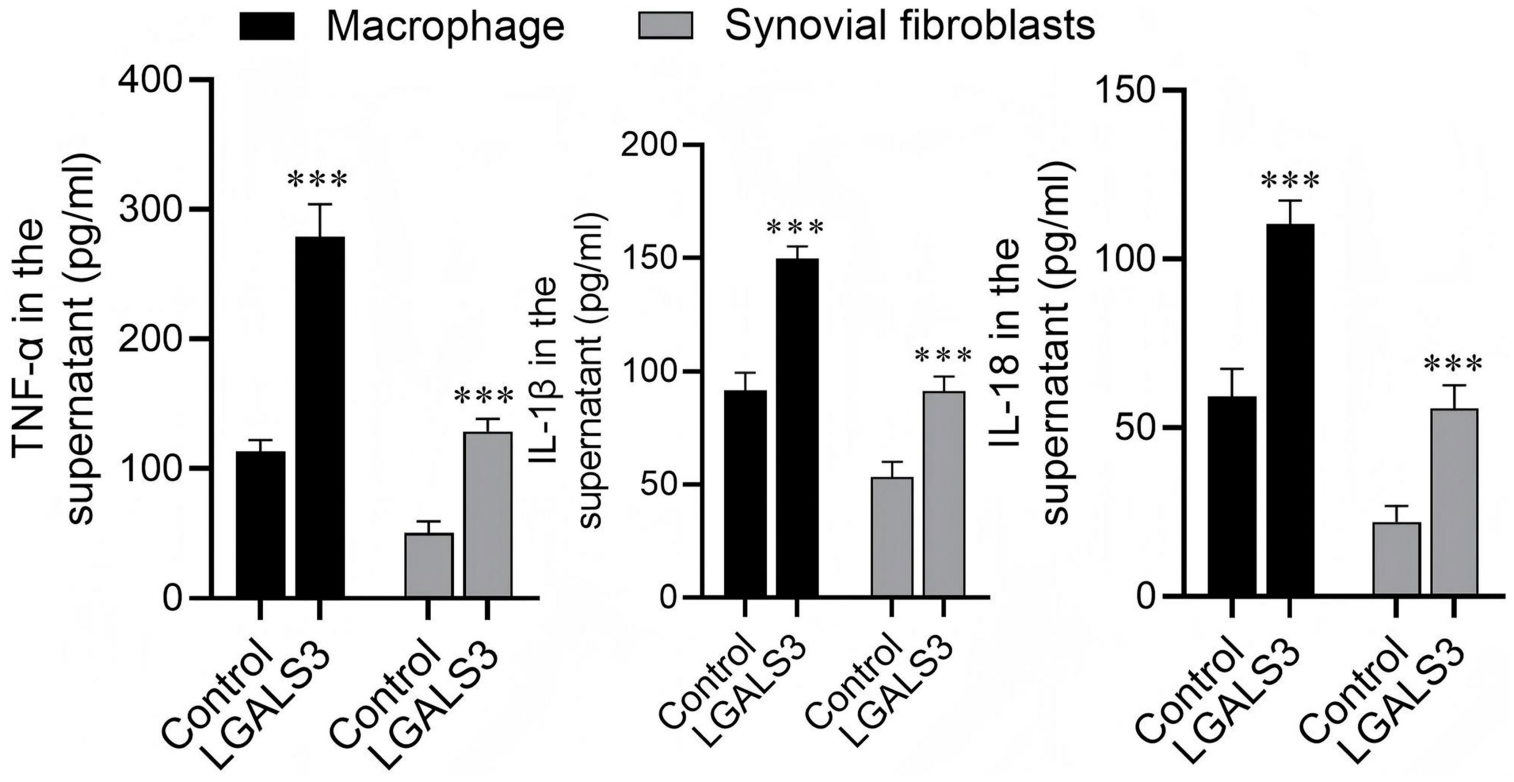
Galectin-3 inducing secretion of pro-inflammatory cytokines in macrophages and synovial fibroblasts.

Compared with the MØ1 and SF1 groups, galectin-3 treatment in the MØ2 and SF2 groups significantly increased the expression of TNF-α, IL-1β, and IL-18 (*p* < 0.05) (Table 3; Figure 6; Supplementary Tables S13–S15). These results collectively suggested that galectin-3 may promote a shift toward a pro-inflammatory state of MØs, providing a cellular basis for its role in KOA-associated inflammation.

**Table 3:** The expression of TNF-α, IL-1β, and IL-18 detected by ELISA.

Group	*n*	IL-1β (mmol/L)	IL-18 (mmol/L)	TNF-α (mmol/L)
Mø1	4	91.507 ± 7.97	59.430 ± 8.09	113.628 ± 8.38
Mø2	4	149.802 ± 5.46	110.399 ± 7.01	279.125 ± 24.89
SF1	4	53.372 ± 6.73	22.199 ± 4.71	50.511 ± 9.08
SF2	4	91.188 ± 6.61	55.864 ± 6.82	128.833 ± 9.59

#### 5.8.3 Modulation of MPO, PRTN3, and P-NF-κB p65 expression by Galectin-3 *in vitro*

**Figure 7:**
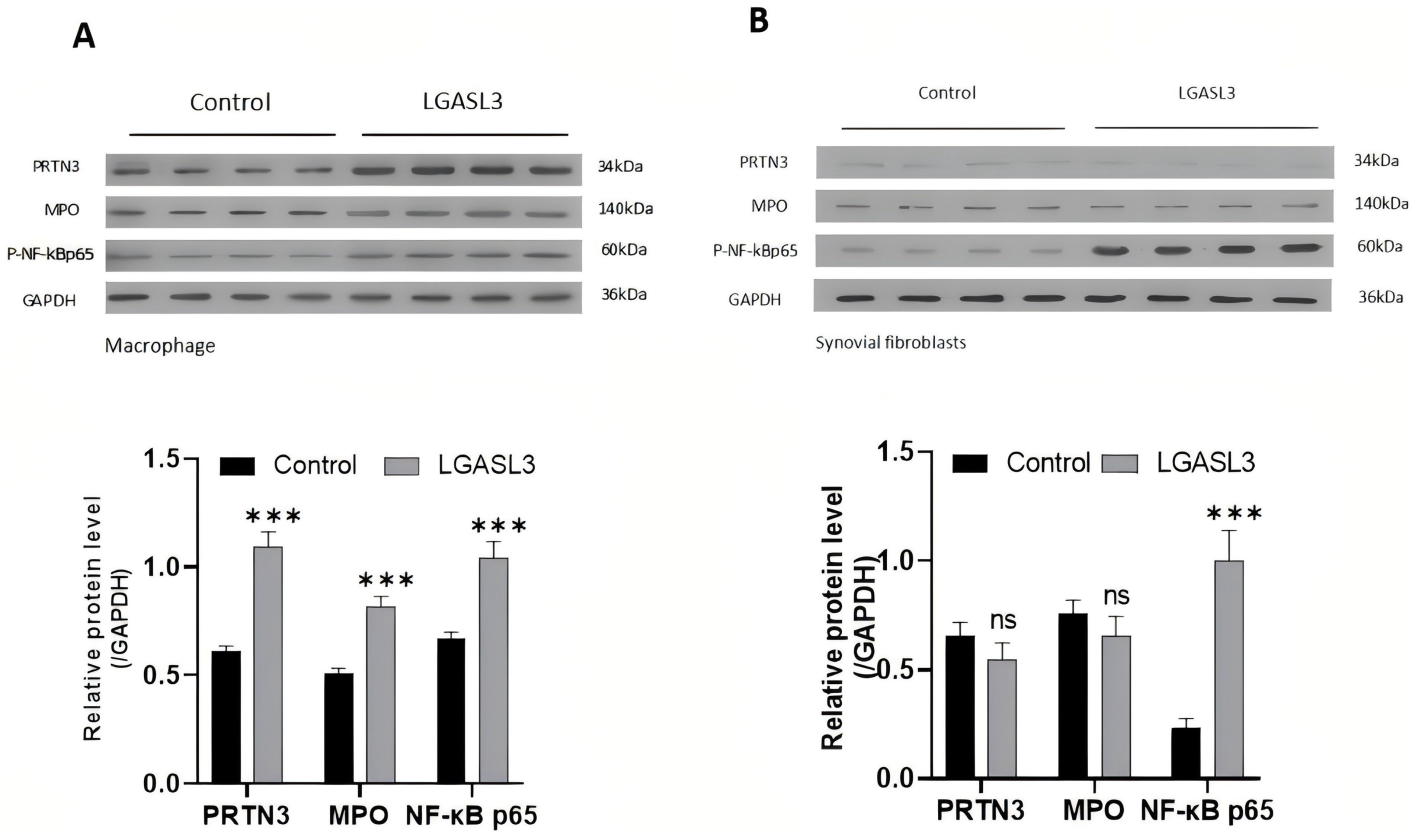
**(A)** Expression of PRTN3/MPO/P-NF-κB p65 in macrophage; **(B)** expression of PRTN3/MPO/P-NF-κB p65 in synovial fibroblasts.

WB results showed that, compared to the MØ1 group, the expression levels of PRTN3, MPO, and P-NF-κB p65 were pronouncedly increased in the MØ2 group (*p* < 0.01; Figure 7A). In contrast, while the SF2 group exhibited a significant upregulation of P-NF-κB p65 expression compared to the SF1 group (*p* < 0.01), no significant differences were observed in the protein expression of PRTN3 or MPO (*p* > 0.05; Figure 7B). This suggested that galectin-3-induced expression of PRTN3 and MPO primarily occurred in MØs, providing initial evidence regarding galectin-3’s regulatory effects on the inflammatory environment associated with KOA.

## 6 Discussion

One challenge in KOA research is the identification of biomarkers genuinely associated with its early pathogenesis. To address this, we employed an integrative approach, combining MR with multidimensional evaluation to systematically identify plasma proteins causally linked with the development of KOA, highlighting galectin-3 as a prioritized candidate.

This prioritization is supported by converging lines of evidence. The MR analysis revealed a marked causal link (OR = 1.07, 95% CI: 1.03–1.11, *p* = 0.00048, FDR-corrected q < 0.1). Moderate co-localization support (PPH4 = 77.3%) suggested a potential shared genetic basis with KOA. Besides, the central position of galectin-3 was found in both the protein–protein interaction network and a key pro-inflammatory KEGG pathway. Furthermore, our exploratory *in vitro* experiments revealed that galectin-3 stimulation appeared to promote pro-inflammatory responses characterized by elevated cytokines (TNF-*α*, IL-1β, IL-18), suggesting a potential association between galectin-3 and the MPO-PRTN3 inflammatory pathway in the pathogenesis of KOA. Collectively, our findings demonstrated the role of galectin-3 not merely as a passive biomarker, but a potential regulatory hub, which warrants further mechanistic and therapeutic exploration in KOA.

### 6.1 Galectin-3 (encoded by LGALS3)

Galectin-3 appears to exert complex, dual effects on KOA pathogenesis, characterized by seemingly contradictory effects across different joint tissues. Evidence has indicated that galectin-3 may exacerbate cartilage degradation by promoting chondrocyte apoptosis and matrix loss via pro-inflammatory pathways such as NF-κB (41). Nevertheless, some studies have observed protective effects of galectin-3 under specific contexts, such as maintaining subchondral bone remodeling homeostasis and supporting chondrocyte survival (42, 43). However, comprehensive evidence has suggested that the net effect of galectin-3 within the microenvironment in KOA may be predominantly pro-inflammatory and pro-degradative (44). This tendency may be attributed to the pronounced pro-inflammatory activity of in galectin-3 synovial MØs, which partially offsets its subtle protective function.

### 6.2 Myeloperoxidase (encoded by MPO)

Myeloperoxidase regulates bone remodeling primarily by modulating reactive oxygen species (ROS)-dependent osteoclast differentiation (45). Previous research has frequently reported downregulated MPO expression in synovial tissues of KOA patients (46), and this low-expression state is believed to induce abnormal intracellular ROS accumulation. Excessive ROS load is speculated to activate key signaling axes such as RANKL–TRAF6–Rac1–NOXs, thereby promoting osteoclast maturation (47). Therefore, the reduced MPO expression observed in our study may exacerbate osteoclast-mediated bone destruction by amplifying downstream signaling activity.

### 6.3 Proteinase 3 (encoded by PRTN3)

Proteinase 3 is recognized as a critical factor linking neutrophil activation with MØ differentiation, both processes central to synovial inflammation in KOA (48). MØs, whose differentiation is driven by proteinase 3, further promote cartilage degeneration and osteophyte formation by releasing inflammatory cytokines and matrix-degrading enzymes (49, 50). Our findings suggested that downregulation of PRTN3 expression may alleviate synovial inflammation and delay KOA progression to some extent by inhibiting MØ differentiation.

### 6.4 Potential pathogenic links between Galectin-3 and joint inflammation in KOA

Multidimensional evidence suggests that galectin-3 may be involved in the regulation of the inflammatory microenvironment in KOA, potentially through modulation of PRTN3 and MPO expression. *In vitro* experiments revealed that this regulatory effect is specific to cell types, with upregulated pro-inflammatory markers observed specifically in synovial MØs following galectin-3 stimulation. Given prior reports linking PRTN3 and MPO to MØ differentiation and subchondral bone remodeling, this finding offers preliminary clues into potential pathological mechanisms (51, 52). The absence of similar responses in SFs likely reflects inherent biological differences among distinct tissue components under pathological conditions, rather than representing conflicting evidence. MR analysis reflects the cumulative impact of lifelong genetic exposure on the risk of a disease, while *in vitro* experiments, as conducted in our study, capture only acute responses to a specific stimulation within 24 h. Overall, these two lines of evidence are complementary, jointly offering an exploratory perspective for understanding the complex pathogenesis of KOA from the dimensions of genetic predisposition and cellular dynamics.

### 6.5 Novelty and clinical implications

Clinically, the causal estimate for galectin-3 (OR = 1.07, 95% CI: 1.03–1.11, *p* = 0.00048) warrants careful interpretation. Although the effect size appears modest, it is important to note that MR reflects the impact of lifelong exposure to genetically determined variations in plasma protein levels, which is distinct from short-term clinical interventions. Even subtle genetic effects may highlight biologically significant pathways. When these pathways are therapeutically targeted, they may yield considerable clinical benefits. Furthermore, in the context of biomarker application, galectin-3 should not be viewed merely as a standalone diagnostic tool, but rather as a mechanistic biomarker. Our co-localization analysis and functional validation support the robustness of its identification, highlighting the specific involvement of the inflammatory pathway mediated by galectin-3 in KOA pathogenesis. This suggests galectin-3 as a promising marker for patient stratification, particularly among individuals with inflammatory phenotypes of KOA, or may be applied in conjunction with other markers to enhance predictive accuracy.

Distinct from previous studies limited to statistical associations, our work integrated proteome-wide MR screening with co-localization analysis and experimental validation. This approach effectively filtered out false positives common in high-throughput screening. Consequently, our study identifies galectin-3 not as a definitive causal factor, but as a prioritized candidate supported by multi-layered evidence, which is readily available for further translational investigation. This workflow offers a robust framework for future discovery of biomarkers in complex osteoarticular diseases.

## 7 Limitations

Although this study identified galectin-3 as a prioritized candidate protein associated with the risk of developing KOA, several limitations must be noted when interpreting these findings.

From the standpoint of genetic statistics, the genetic data used in this study are derived solely from European populations, limiting the generalizability of our findings to other ethnic populations. Furthermore, our study heavily relies on public databases. Despite employing multiple validation approaches, it may still be constrained by potential delays in database updates.

In terms of *in vitro* validation, only acute cellular responses within 24 h of stimulation with a single concentration of galectin-3 are captured, which cannot fully reflect the prolonged and complex pathological progression of KOA. Most importantly, due to the absence of loss-of-function experiments, such as small interfering RNA (siRNA) knockdown or neutralizing antibody blockade, the potential associations between LGALS3 and MPO or PRTN3 currently observed should be regarded as preliminary working hypotheses rather than a confirmed regulatory axis. Additionally, our study is limited to MØs and SFs. Therefore, these associations remain to be investigated in chondrocytes or animal models.

To further explore the potential link between galectin-3 and KOA, we have planned future research directions. These include rigorous dose–response analyses and loss-of-function experiments to clarify the potential associations of LGALS3 with MPO and PRTN3. We also plan to expand our investigation to include chondrocyte-driven matrix metabolism, aiming to construct a more comprehensive molecular map of the disease. Additionally, validation in *in vivo* animal models and longitudinal clinical cohorts is also planned to explore the clinical value of galectin-3 as a therapeutic target for KOA.

## 8 Conclusion

In conclusion, our comprehensive plasma proteome-wide MR study, augmented by PPI network analysis, identifies galectin-3 as a promising candidate protein associated with KOA, and this association was replicated in an independent FinnGen cohort. The findings offer preliminary insights into the role of galectin-3 in KOA pathogenesis by proposing a working hypothesis of the pro-inflammatory LGALS3-MPO-PRTN3 pathway. This potential link is consistent with our exploratory *in vitro* findings, which reveal that galectin-3 stimulation upregulates inflammatory cytokines (TNF-*α*, IL-1β, IL-18) and is associated with increased PRTN3/MPO expression in MØs, while predominantly activating NF-κB in SFs. By integrating large-scale genetic databases, uncovering PPI-driven pathways, and observing preliminary cellular dynamics, our work provides suggestive etiological clues into KOA and identifies galectin-3 as a potential plasma biomarker. The findings suggest that the LGALS3-MPO-PRTN3 pathway may represent a candidate target for therapeutic intervention, which warrants further mechanistic confirmation.

## Data Availability

The original contributions presented in the study are included in the article/Supplementary material, further inquiries can be directed to the corresponding author.

## Footnotes

1
https://opengwas.io/datasets/

2
https://www.ncbi.nlm.nih.gov/

3
https://string-db.org
