# Correction: Identification of novel protein biomarkers for knee osteoarthritis by integrating human plasma proteome: evidence from Mendelian randomization and preliminary *in vitro* investigation

**DOI:** 10.3389/fmed.2026.1926672

**Published:** 2026-07-15

**Authors:** Wang Huang, WeiWei Hu, JiaYu Chen, Dan Yuan

**Affiliations:** 1 Qiannan Prefecture Traditional Chinese Medicine Hospital, Duyun, Guizhou, China; 2 Department of Orthopedics and Traumatology, Pingjiang County Hospital of Traditional Chinese Medicine, Yueyang, Hunan, China

**Keywords:** biomarkers, causal effect, knee osteoarthritis, Mendelian randomization, plasma protein

An incorrect **Funding** statement was provided. The **Funding** statement is “The author(s) declared that financial support was not received for this work and/or its publication.”

The original version of this article has been updated.

