# Supplementary figures and images for: Identification of novel protein biomarkers for knee osteoarthritis by integrating human plasma proteome: evidence from Mendelian randomization and preliminary in vitro investigation
Source: Front Med (Lausanne). 2026 Apr 22;13:1745114. doi: 10.3389/fmed.2026.1745114 (PMC13143750; doi:10.3389/fmed.2026.1745114)

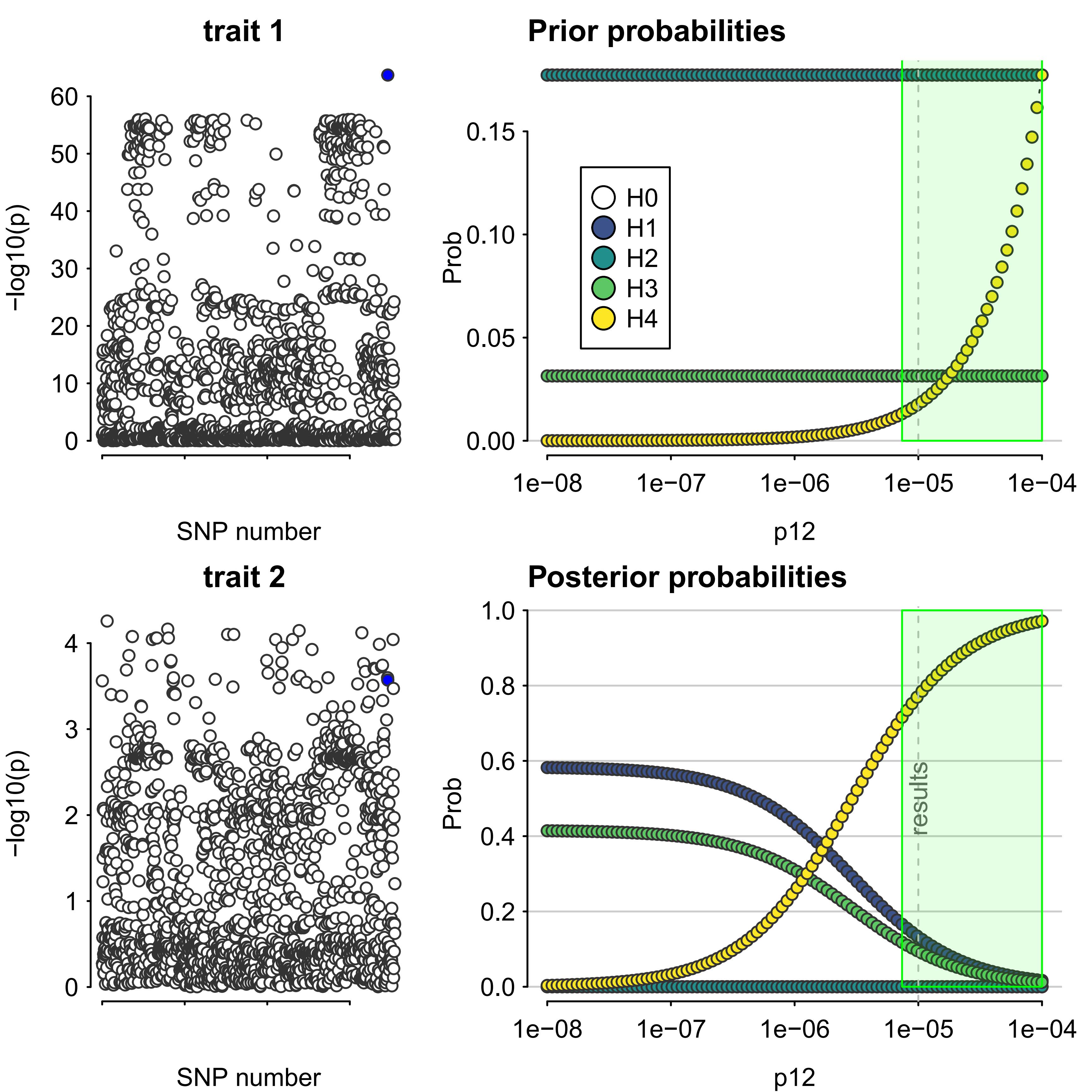

Supplement: Supplementary file 2 [file Image_1.JPEG]

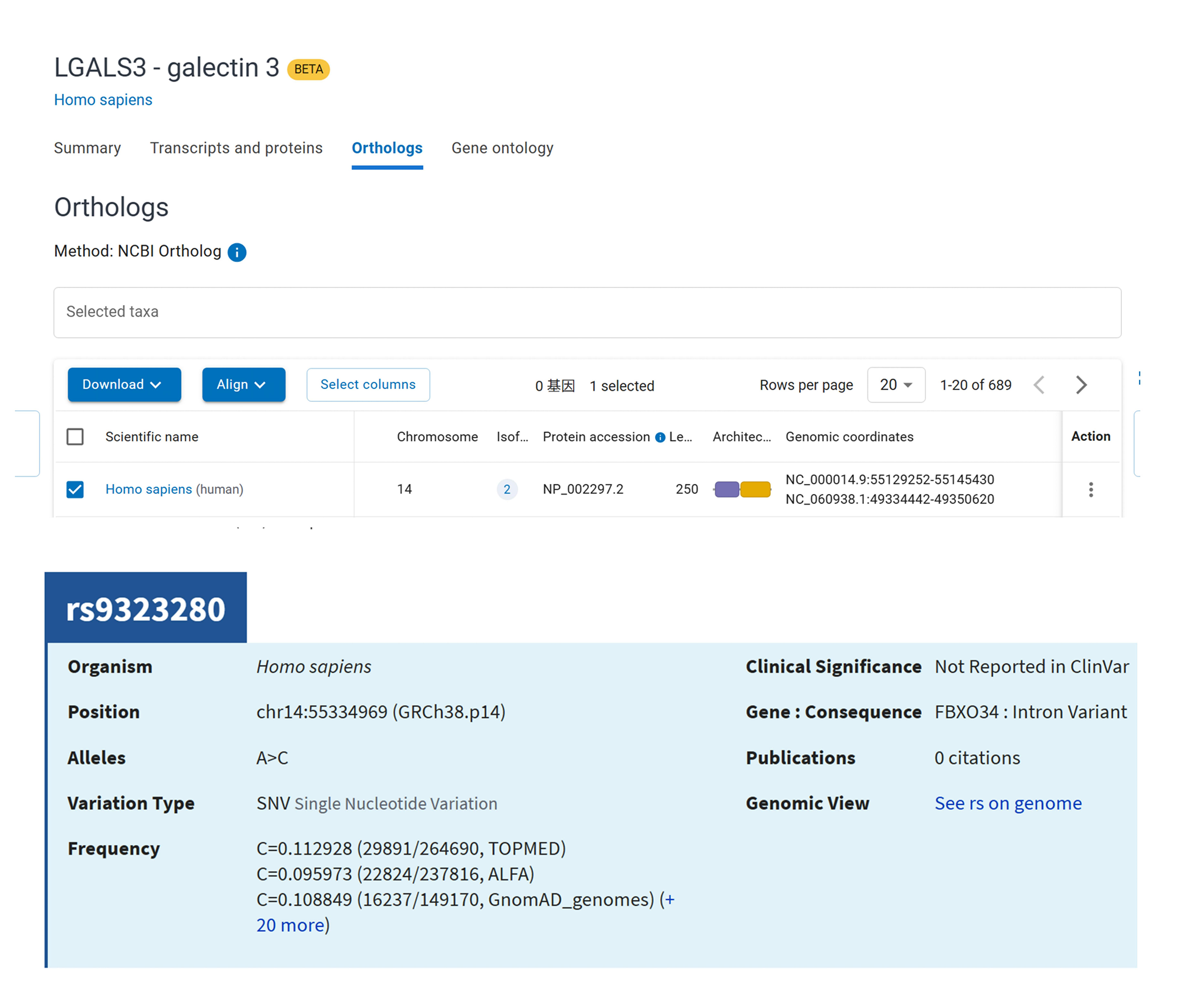

Supplement: Supplementary file 3 [file Image_2.TIF]

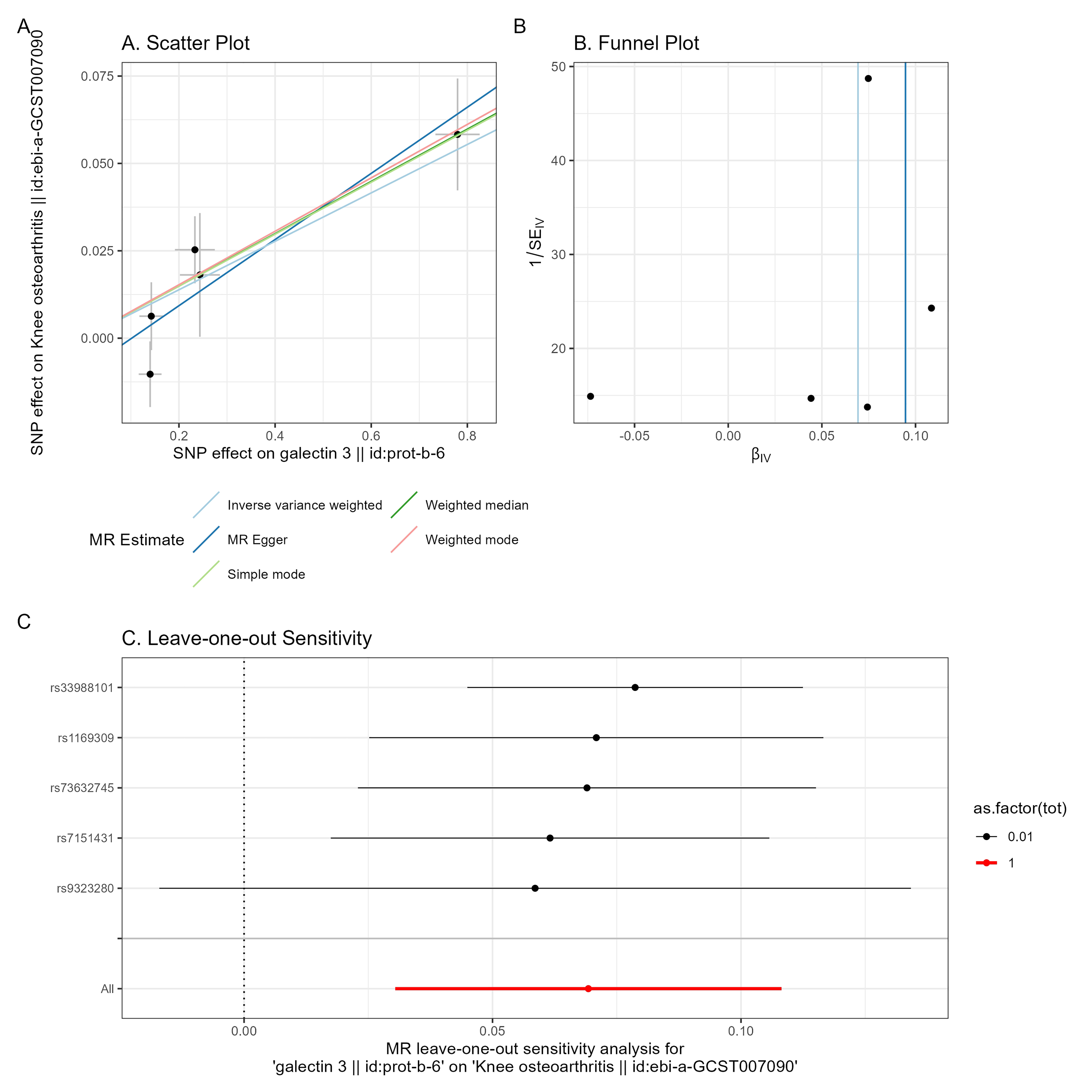

Supplement: Supplementary file 4 [file Image_3.TIF]
